# C-reactive protein and coronary atheroma regression following statin therapy: A meta-regression of randomized controlled trials

**DOI:** 10.3389/fcvm.2022.989527

**Published:** 2022-11-11

**Authors:** Darui Gao, Rong Hua, Dina Jiesisibieke, Yanjun Ma, Chenglong Li, Sijing Wu, Qian Ma, Wuxiang Xie

**Affiliations:** ^1^Peking University Clinical Research Institute, Peking University First Hospital, Beijing, China; ^2^Peking University Clinical Research Institute Heart and Vascular Health Research Center at Peking University Shougang Hospital, Beijing, China; ^3^Key Laboratory of Molecular Cardiovascular Sciences (Peking University), Ministry of Education, Beijing, China; ^4^Peking University Third Hospital, Beijing, China; ^5^Department of Cardiology, Beijing Anzhen Hospital, Capital Medical University, Beijing, China

**Keywords:** statins, regression of atherosclerosis, C-reactive protein, randomized controlled trial, meta-analysis

## Abstract

**Objective:**

Several clinical trials have indicated that statins stabilize and reverse atherosclerotic plaque. However, different studies have provided inconsistent findings regarding mechanisms and influencing factors of plaque regression under statin therapy. Apart from lipid-lowering effect, statins have pleiotropic effects including anti inflammation in humans. In this study, meta-analysis and meta-regression were used to determine the effects of statin medications on coronary plaque volume. Meanwhile, to assess whether statins promote plaque regression effect was related to their anti-inflammatory ability, the impact of CRP/hsCRP reduction during statin therapy on plaque regression was investigated.

**Methods:**

Up to June 15, 2022, a systematic PubMed, EMBASE, and Cochrane search was performed for randomized controlled trials that assessed treatment effect using total atheroma volume (TAV), percent atheroma volume (PAV), or plaque volume (PV). Only CRP/hsCRP and LDL-C values reported before and after treatment were considered.

**Results:**

12 studies (2,812 patients with heart and/or vascular disease) fulfilled the inclusion criteria and were included in the systematic review. A meta-analysis of 15 statin-treated arms reported a significant reduction in change of TAV/PV [standardized mean difference (SMD): –0.27, 95% confidence intervals (–CI): –0.42, –0.12, *p* < 0.001], compared with the control arms. Another meta-analysis of 7 trials also found that patients in the intervention group had a significant reduction in change of PAV (SMD: -0.16, 95% CI: –0.29, –0.03, *p* = 0.019), compared with those in the control group. Meta-regressionanalysis revealed that the percent change of CRP/hsCRP was significantly associated with SMD in change of TAV/PV after adjusting for percent change of LDL-C, age, gender and study duration. Meta-regression analysis showed that percent change of CRP/hsCRP statistically influenced SMD in change of PAV, when percent change of CRP/hsCRP was included separately. However, the percent change of CRP/hsCRP was not significantly associated with SMD of PAV change after adjusting for all covariates.

**Conclusion:**

In conclusion, statin therapy is beneficial for plaque regression. Statins promote plaque regression, which might be associated to their anti-inflammatory ability.

## Introduction

Cardiovascular diseases are considered the leading causes of death worldwide. Among them, coronary heart disease (CHD) has garnered considerable attention due to its high prevalence and burden. The pathological basis of CHD is atherosclerosis, which is characterized by the accumulation of lipids and cholesterol in the artery’s subintima and progressive chronic inflammation of the fibrotic plaque on the wall of great and medium arteries (1). Assessment of coronary artery plaques provides clinical information regarding the progression of disease and the risk of experiencing future adverse cardiovascular events (2). In recent studies, indicators including total atheroma volume (TAV), percent atheroma volume (PAV), or plaque volume (PV) have been widely used to assess plaque burden (3).

Coronary plaque regression has a significant positive correlation with low density lipoprotein cholesterol (LDL-C). As important lipid-lowering drugs, several studies have demonstrated that statin drugs promote coronary atheroma stabilization and regression in patients with acute coronary events or stable coronary disease (4). Among those studies, recent clinical studies have demonstrated that statins can reduce plaque burden by demonstrating a reduction in TAV, PAV, and PV (5). Currently, statins are widely used to prevent atherosclerotic cardiovascular disease (ASCVD). Numerous studies have shown that statins are effective in reducing LDL-C, and the risk of death and recurrent coronary and cardiovascular events in those with a history of ASCVD (6). Meanwhile, statin therapy is a first-line treatment for the primary prevention of ASCVD in patients with elevated low-density lipoprotein cholesterol levels (≥ 190 mg/dL), those with diabetes mellitus, those who are 40–75 years of age, and those determined to be at sufficient ASCVD risk after a clinician–patient risk discussion (7).

As the mechanism of vascular inflammation is gradually elucidated, numerous evidences have demonstrated that C-reactive protein (CRP) and high-sensitivity C-reactive protein (hsCRP) may play direct pathogenic roles in atherosclerosis (8, 9). Initially, statin drugs were used primarily to reduce blood lipids. With the deepening of research, its non-lipid-lowering effects, such as the anti-inflammatory effect of statins on the coronary plaque volume, have become the focus of recent studies. Ridker et al. discovered that rosuvastatin (20 mg/d) and placebo were administered to randomly selected healthy people with elevated hs-CRP but no evidence of hyperlipidemia. After an average follow-up of 1.9 years, the hs-CRP level in the treatment group decreased by 37% compared with that in the control group, implying that statins may have anti-atherosclerosis functions *via* anti-inflammatory mechanisms (10). Numerous clinical trials, such as the Air Force/Texas Coronary Atherosclerosis Prevention (AFCAPS/TexCAPS) study, the Reversal of Atherosclerosis with Aggressive Lipid Lowering (REVERSAL) trial, and the Pravastatin or Atorvastatin Evaluation and Infection Therapy-Thrombolysis In Myocardial Infarction 22 (PROVE IT-TIMI 22) trial, have demonstrated that statins reduce hsCRP levels independently of lowering LDL-C levels. In a trial with canakinumab for atherosclerotic disease, the rate of cardiovascular event recurrence was significantly lower in the treated group than in the placebo group, implying that reducing inflammation without affecting lipid levels can reduce cardiovascular disease risk (11).

Statin therapy was shown to be beneficial in reducing CRP/hsCRP. However, few studies have attempted to investigate the relationship between the degree of CRP/hsCRP reduction associated with changes in coronary plaque burden during statins treatment. To answer the question of whether the CRP/hsCRP lowering effect of statins could delay or reverse the progression of atherosclerosis, we conducted this study. The aim of the present study was to provide a systematic review and meta-regression analysis to examine the impact of statins on CRP/hsCRP reduction on coronary plaque burden assessed with TAV, PAV, and PV. At the same time, we analyzed the joint effects of LDL-C and CRP/hsCRP changes on plaques.

## Methods

This work followed the Preferred Reporting Items for Systematic reviews and Meta-Analyses (PRISMA) and amendments to the Quality of Reporting of Meta-analyses (QUOROM) statement (12, 13).

## Search strategy and study selection

For this meta-analysis, we conducted a search in PubMed, EMBASE and the Cochrane Library to identify studies relevant to this topic from their inception to June 15, 2022. The study selection was performed independently by 2-group investigators (CLL, YJM as group 1, and RH, DJ as group 2) using highly sensitive strategy. Disagreements were resolved by consensus with a senior author (WXX). Here we show the search strategy of PubMed: “[(statin) OR (hydroxy-methyl-glutaryl-CoA) OR (HMG-COA) OR (pravastatin) OR (lovastatin) OR (simvastatin) OR (Atorvastatin) OR (fluvastatin) OR (Rosuvastatin) OR (Pitavastatin)] AND [(intravascular ultrasound) OR (IVUS) OR (plaque) OR (atheroma)] AND [(intravascular ultrasound) OR (IVUS) OR (coronary)] AND (Clinical Trial[ptyp]).” Supplementary Table 1 shows details of the search syntax.

## Selection criteria

Studies were included according to the following criteria: (a) randomized controlled trials (RCTs); (b) investigating the impact of statin therapy on plaque volume using IVUS; (c) reporting at least one of the following data: TAV, PV, and PAV; (d) with a follow-up longer than or equal to 6 months; (e) reporting LDL-C at baseline and the end of the study or reporting data of percent change of LDL-C; (d) reporting CRP or hsCRP before and after statin treatment (or percent change of CRP/hs-CRP).

Exclusion criteria included the following: (a) duplicate publication or secondary analyses of the same study population; (b) lack of sufficient information on baseline or follow-up IVUS data, LDL-C data, and CRP/hsCRP data.

## Data extraction quality appraisal

The data were extracted from each study using standard tables. The extracted data included the following: study characteristics (the first author, title, publication time, number of patients, country, and study duration), patient characteristics (age and sex), intervention, control, method characteristics (randomization, blind implementation, and follow-up loss), and patient outcomes. For patient outcomes, we extracted TAV, PAV, or PV data as measured using IVUS technique, LDL-C data, CRP, hsCRP data (including values at baseline and endpoint) and other useful information.

After data extraction, we conducted statistical analysis to calculate change of TAV, change of PV, change of PAV, percent change of LDL-C, percent change of CRP, and percent change of hsCRP. Articles reported mean values and standard deviation (SD) of change of TAV/PV/PAV, the original number was entered. Some studies (14–17) did not report SD values, which were filled by using the SD of the baseline data of the control group. 1 study (18) provided standard error (SE) rather than SD, and then SD value was calculated based on SE value. If the IVUS efficacy endpoints were reported as medians, with distribution-free 95% confidence intervals (CI), the median reported in the original text was extracted, and SD was calculated by formula.

In terms of LDL-C, if the article reported percent change of LDL-C, the original number was entered; otherwise, percent change of LDL-C was calculated using the following formula:


$$percentchangeofLDL-C(\%)=\frac{followupvalue-baselinevalue}{baselinevalue}\times 100\%$$

Percent change of CRP and percent change of hsCRP were calculated using the same approach. Supplementary Table 2 shows details of data extraction.

Two independent authors (RH and DRG) assessed the risk of bias in each included study. According to Cochrane’s indications, un-blinded, independent reviewers evaluated the quality of included studies using pre-specified forms (risk of bias table), including seven examined fields: random sequence generation (selection bias); allocation sequence concealment (selection bias); blinding of participants and personnel (performance bias); blinding of outcome assessment (detection bias); incomplete outcome data (attrition bias); selective outcome reporting (reporting bias); and other potential sources of bias.

## Data analysis and synthesis

Continuous variables were expressed as mean ± *SD*, whereas categorical variables were expressed as *n* (%). Heterogeneity among individual studies was assessed with the Q-test and quantified with the *I*^2^ statistic (range: 0–100%). *I*^2^ represents the proportion of the total variance that can be attributed to heterogeneity of true study effects (19). The heterogeneity was regarded as low if *I*^2^ ≤ 25%, as moderate if *I*^2^ in the range of 26–74% and as high if *I*^2^ ≥ 75% (20). When a study is gathered from the published literature, the random-effects model is generally a more plausible match. For the random-effects model allows the true effect size may vary from study to study. In addition, the standard error of the summary effect and the confidence intervals for the summary effect are wider under the random-effects model than under the fixed-effect model (21). Thus, we performed meta-analysis to pool estimates using random effects model. Meta-analysis with continuous outcome variables was performed, and the effect of statin therapy (vs. control) on change of TAV, PV, and PAV at the end of follow-up was estimated as standardized mean difference (SMD) and 95% CI. If *p* < 0.05 and the 95% CI did not include zero, the point estimate of SMD was considered statistically significant. To avoid double-counting of subjects and consequent unit-of-analysis error in trials with more than one treatment arm, the control group was evenly divided (where possible) (10). Since the units (mm^3^) of change of TAV and change of PV were the same, we combined these two indicators for data synthesis.

To explore the link between the dependent variable and the covariate, meta-regression is often used. We hypothesized that the included studies may have shown differences according to the percent change of CRP/hsCRP, percent change of LDL-C, age, gender and study duration of the patients. To evaluate the possible impact of these factors on the results of the meta-analysis, we established model with the change of TAV/PV or change of PAV as the dependent variable. In particular, change in TAV/PV was our primary outcome, and change in PAV was the secondary outcome.

Funnel plot analysis and Begg’s and Egger’s tests were performed to evaluate potential publication bias. Sensitivity analysis was conducted to assess the stability of studies. Sensitivity analysis was conducted using leave-one-out method, i.e., removing one study each time and repeating the analysis. Statistical analyses were carried out using meta packages in *R* version 4.1.2 (2021-11-01) and risk of bias was evaluated with Review Manager (RevMan 5.3; Cochrane Collaboration).

## Result

### Flowchart of included studies

**FIGURE 1:**
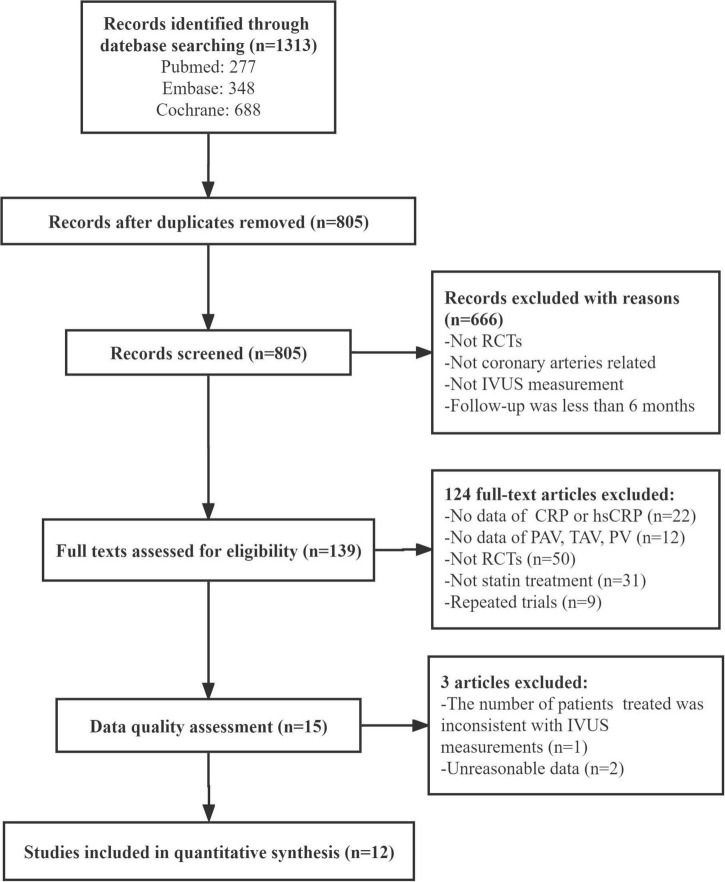
Flowchart for study. RCTs, randomized controlled trials; IVUS, intravenous ultrasound; CRP, C-reactive protein; hsCRP, high-sensitivity C-reactive protein; TAV, total atheroma volume; PAV, percent atheroma volume; PV, plaque volume.

The initial literature search retrieved 1,313 articles. After the removal of duplicates, the titles and abstracts of 805 articles were carefully checked, leading to the exclusion of 666 articles for failing to meet the inclusion criteria. Initially, 139 articles were selected, and their full texts were evaluated. Of them, 124 articles were excluded: 22 because CRP/hsCRP levels were not reported, 12 because plaque evaluation (TAV, PAV, or PV) was not performed, 50 because they were not RCTs, 31 because statins were not used, and 9 because of repeated trials. A total of 15 articles entered the third round of evaluation. One was excluded due to a discrepancy between the number of participants receiving statins and the number of people participating in IVUS measurements (22). And two were excluded because of data quality: in one study, CRP was reported, but the indicators of the control group declined significantly (23); in another study, the *SD* at baseline and follow-up varied greatly and the reported difference value was inconsistent with the calculated difference value (24). Overall, this analysis included 12 trials (14–18, 25–31) Figure 1 summarizes the study selection process.

### Characteristics of included studies

The study characteristics are reported in Table 1. A total of 2,812 subjects were included in the 12 eligible studies. Included studies were published between 2004 and 2016 and were reported from China, the USA, Korea and Japan. The largest study had a population size of 1,039 subjects while the smallest study recruited 30 subjects. The mean age of the participants ranged from 55.8 to 67.0 years.

**TABLE 1:** Main characteristics and findings of included studies.

References	Country	Study duration	Therapy^a^ (mg/d)	Participants (*n*)	Age (years)	Male (%)	CRP/hsCRP	Percent change of CRP/hsCRP (%)	Percent change of LDL-C (%)	Change of TAV/PV (mm^3^)	Change of PAV (%)
Hong et al. (27)	Korea	12 months	Ros 20	16	60 ± 8	75	hsCRP	–94.35	–46.38	–5.62 ± 7.71	–0.80 ± 1.27
			A to 40	14	62 ± 90	43		–93.85	–43.31	–4.74 ± 8.51	–0.57 ± 1.15
Hong et al. (28)	Korea	11 months	Ros 20	65	59 ± 10	75	hsCRP	–80	–49.18	–4.4 ± 7.3	–0.73 ± 2.05
			A to 40	63	58 ± 10	73		–89.25	–40.17	–3.6 ± 6.8	–0.19 ± 2.10
Kawasaki et al. (15)	Japan	6 months	A to 20	18	66 ± 8.7	70.6	CRP	–65	–39	–3.8 ± 32.2	/
			Pra 20	17	67 ± 7.8	72.2		–18	–32	–1.6 ± 32.1	/
			Diet	17	66 ± 6.4	82.4		17	–2	0 ± 29.9	/
Nicholls et al. (29)	The USA, et al	26 months	Ros 40	520	57.4 ± 8.6	72.9	CRP	–35.29	–47.83	–6.39 ± 13.96	–1.22 ± 3.61
			A to 80	519	57.9 ± 8.5	74.4		–33.33	–41.45	–4.42 ± 15.81	–0.99 ± 3.49
Nissen et al. (30)	The USA	18 months	A to 80	253	55.8 ± 9.8	71	CRP	–36.4	–46.3	–0.9 ± 20.69	0.2 ± 3.25
			Pra 40	249	56.6 ± 9.2	73		–5.2	–25.2	4.4 ± 23.75	1.6 ± 4.03
Nozue et al. (31)	Japan	8 months	Pit 4	58	66 ± 9	90	hsCRP	–75	–41	/	–0.2 ± 3.4
			Pra 20	61	67 ± 11	77		–75	–29	/	0.2 ± 4.8
Park et al. (18)	Korea	12 months	Ros 40	152	62.6 ± 9.3	71	hsCRP	–52.38	–43.87	–14.72 ± 29.59	–0.88 ± 4.93
			Ros 10	73	61.8 ± 8.9	77		–47.83	–27.90	–13.63 ± 21.87	–0.85 ± 3.25
Takayama et al. (16)	Japan	12 months	Ros 20	18	65.1 ± 10.1	72	hsCRP	–65	–50	–3.1 ± 33.5	/
			Ros 2.5	19	63.8 ± 8.5	83		–60	–30	1.2 ± 33.5	/
Hiro et al. (25)	Japan	8–12 months	A to 20	127	62.4 ± 10.6	81.1	hsCRP	–95.4	–35.8	–10.6 ± 10.6	–6.3 ± 6.1
			Pit 4	125	62.5 ± 11.5	82.4		–97.3	–36.2	–8.2 ± 8.9	–5.7 ± 6.3
Hong et al. (26)	Korea	12 months	Ros 10	50	59 ± 9	74	CRP	–57.14	–44.83	–3.6 ± 7.2	/
			Sim 20	50	58 ± 10	80		–29.41	–34.45	–1.8 ± 5.7	/
Zhang et al. (17)	China	9 months	A to 80	50	64.5 ± 13.8	62	hsCRP	–66.36	–40.91	–1.5 ± 9.33	/
			A to 20	50	65.5 ± 6.2	58		–37.41	–24.58	8.36 ± 9.33	/
Guo et al. (14)	China	6 months	A to 10	47	62.64 ± 12.00	85.1	hsCRP	11.59	–22.11	–0.02 ± 13.76	/
			A to 20	45	59.18 ± 8.48	80.0		0.39	–31.16	2.29 ± 13.76	/
			A to 40	43	58.91 ± 12.90	95.3		–13.94	–36.21	–6.37 ± 13.76	/
			A to 80	39	58.95 ± 9.68	87.2		–41.15	–36.04	–11.48 ± 13.76	/
			Placebo	54	62.07 ± 8.51	88.9		35.50	1.02	2.63 ± 13.76	/

*^a^*Ros, rosuvastatin; Ato, atorvastatin; Pra, pravastatin; Pit, pitavastatin; Sim, simvastatin.

12 trials with 16 treatment arms were included. 8 treatment arms used atorvastatin (dose range: 10–80 mg/day; duration of treatment: 24–72 weeks), 6 treatment arms used rosuvastatin (dose range: 10–40 mg/day; duration of treatment: 44–104 weeks), 1 treatment arm used pravastatin (dose: 20 mg/day; duration of treatment: 24 weeks), and 1 treatment arm used pitavastatin (dose: 4 mg/day; duration of treatment: 32 weeks).

IVUS was used in all studies to evaluate plaque volume. In addition to 1 study (24) 11 studies reported change of TAV/PV, and 7 studies reported change of PAV. As described in the data extraction section, percent change of CRP/hsCRP and percent change of LDL-C were reported in all studies.

Overall, random sequence generation was observed in 6 studies, 4 of them reported allocation concealment. 3 trials were double-blinded, and 8 studies performed blinded assessments of the outcomes. Moreover, 2 studies existed incomplete outcome data because of a high attrition rate. Supplementary Figure 1 shows details of the risk of bias assessment.

### Effect of statin therapy on change of TAV/PV

**FIGURE 2:**
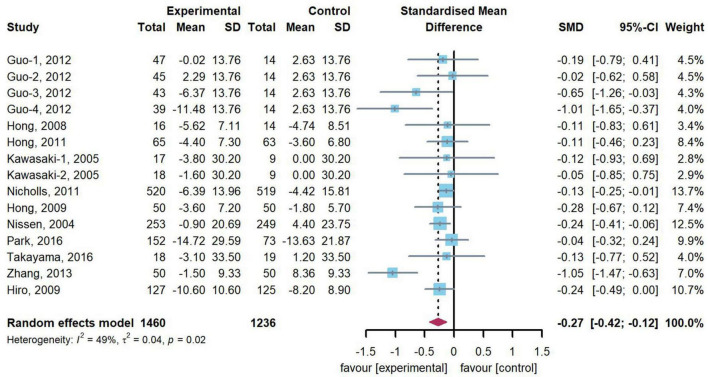
Forest plot of change of TAV/PV. A meta-analysis of 15 statin-treated arms reported a significant reduction in change of TAV/PV [standardized mean difference (SMD): –0.27, 95% confidence intervals (CI): –0.42, –0.12], compared with the control arms.

11 trials (*n* = 2,696) including 15 comparisons reported change of TAV/PV. Compared with control arms, our meta-analysis showed that 15 treatment arms revealed a significant decrease in change of TAV/PV (SMD: –0.27, 95% CI: –0.42, –0.12, *p* < 0.001), with a moderate heterogeneity (*Q* = 27.55, *df* = 17, *p* = 0.02, *I*^2^ = 49.2%). Figure 2 presents the combined results of the 15 head-to-head comparisons in this meta-analysis.

### Effect of statin therapy on change of percent atheroma volume

**FIGURE 3:**
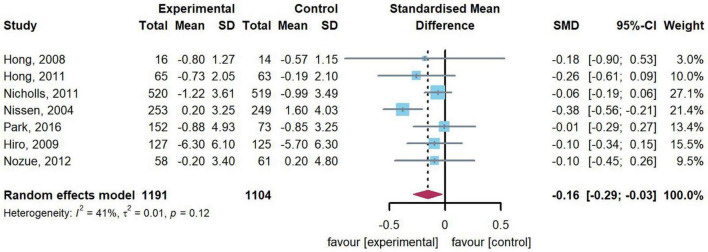
Forest plot of change of PAV. A meta-analysis of 7 studies reported a significant reduction in change of PAV [standardized mean difference (SMD): –0.16, 95% confidence intervals (CI): –0.29, –0.03], compared with the control.

7 studies (*n* = 2,295) reported change of PAV. Heterogeneity test of data from 7 studies shown moderate heterogeneity (*Q* = 10.19, *df* = 6, *p* = 0.12, *I*^2^ = 41.1%) and random effect model was adopted. Compared with those in the control group, this meta-analysis indicated that patients in the intervention group have a significant reduction in change of PAV (SMD: –0.16, 95% CI: –0.29, –0.03, *p* = 0.019). Figure 3 presents the combined results of 7 studies in this meta-analysis.

### Meta-regression for standardized mean difference in change of TAV/PV

Meta-regression was then employed to test whether the percent change of CRP/hsCRP was associated with the change of TAV/PV. The results of the meta-regression analysis are given in Table 2. Model 1 demonstrates that the impact of percent change of CRP/hsCRP on change of TAV/PV was statistically significant (*p* = 0.024). The regression coefficient of this independent variable was β = 0.0064 (95% CI: 0.0009–0.0120). Model 2 analyzed the influence of percent change of LDL-C on change of TAV/PV. The results showed that percent change of LDL-C had no significant effect on change of TAV/PV (*p* = 0.268). Model 3 incorporates percent changes of CRP/hsCRP and LDL-C. Only percent change of CRP/hsCRP was associated with change of TAV/PV (β = 0.0119, 95% CI: 0.0017–0.0221, *p* = 0.022). In Model 4, we entered percent change of CRP/hsCRP, percent change of LDL-C, age, gender and study duration. Among them, only percent change of CRP/hsCRP statistically influenced the dependent variable (*p* = 0.046).

**TABLE 2:** Meta-regression analysis for SMD in change of TAV/PV.

Variables	Model 1	Model 2	Model 3	Model 4
	β (95% CI)	β (95% CI)	β (95% CI)	β (95% CI)
Intercept	–0.1463 (–0.2982, 0.0057)	–0.1513 (–0.4122, 0.1095)	–0.2419* (–0.4510, –0.0329)	–0.6063 (–5.5278, 4.3152)
Percent change of CRP/hsCRP^a^	0.0064* (0.0009, 0.0120)	–	0.0119* (0.0017, 0.0221)	0.0116* (0.0002, 0.0230)
Percent change of LDL–C	–	0.0075 (–0.0058, 0.0208)	–0.0129 (–0.0333, 0.0075)	–0.0135 (–0.0375, 0.0104)
Age	–	–	–	–0.0057 (–0.0690, 0.0577)
Gender	–	–	–	0.0074 (–0.0133, 0.0281)
Study duration	–	–	–	0.0092 (–0.0237, 0.0421)

^a^ *p < 0.05.

### Meta-regression for standardized mean difference in change of percent atheroma volume

Similarly, we performed another meta-regression to explore how the percent change of CRP/hsCRP affects change of PAV. The results of the meta-regression analysis are given in Table 3. Model 1 used the percent change of CRP/hsCRP as an independent variable. The results indicated that the percent change of CRP/hsCRP (β = 0.0086, 95% CI: 0.0022–0.0150) affects PAV change (*p* = 0.009). When the percent change of CRP/hsCRP was higher, change of PAV was greater. Model 2 shows that the percent change of LDL-C was not significantly associated with PAV change (*p* = 0.066). In Model 3 (both percent change of CRP/hsCRP and percent change of LDL-C were included as independent variables) and Model 4 (independent variables including percent change of CRP/hsCRP, percent change of LDL-C, age, gender and study duration), multivariable meta-regression analyses did not reveal any significance between independent variables and the change of PAV.

**TABLE 3:** Meta-regression analysis for SMD in change of PAV.

Variables	Model 1	Model 2	Model 3	Model 4
	β (95% CI)	β (95% CI)	β (95% CI)	β (95% CI)
Intercept	–0.0833 (0.1784, 0.0117)	–0.0217 (–0.1926, 0.1493)	-0.0735 (–0.2504, 0.1034)	-2.6636 (–6.5456, 1.2183)
Percent change of CRP/hsCRP^a^	0.0086** (0.0022, 0.0150)	–	0.0079 (–0.0043, 0.0201)	0.0039 (–0.0113, 0.0190)
Percent change of LDL-C	–	0.0127 (–0.0008, 0.0261)	0.0015 (–0.0209, 0.0238)	0.0010 (–0.0256, 0.0277)
Age	–	–	–	0.0458 (–0.0213, 0.1129)
Gender	–	–	–	–0.0060 (–0.0405, 0.0285)
Study duration	–	–	–	0.0160 (–0.0051, 0.0371)

^a^ **p < 0.01.

### Publication bias and sensitivity analysis

Although Begg’s rank correlation (*p* = 0.7290) and Egger’s linear regression (*p* = 0.2323) tests were not significant, the funnel plot was asymmetric, implying potential publication bias in reporting the effect of statin therapy on change of TAV/PV. Regarding the impact of statin therapy on change of PAV, the number of studies was insufficient to conduct Begg’s test and Egger’s tests. However, the funnel plot also indicated potential publication bias. Funnel plots are presented in Supplementary Figures 2, 3.

Sensitivity analysis by excluding one study each time confirmed that the pooled estimate was consistent among studies with balanced weight. Additional sensitivity analyses are presented in Supplementary Figures 4, 5.

## Discussion

This meta-analysis comprised RCTs using IVUS to measure coronary plaque burden and reporting results of TAV, PAV, or PV changes. The present meta-analysis demonstrated that (1) quantitative synthesis revealed a decrease in TAV/PV and PAV levels after statin treatment compared with the control. All studies included in the meta-analysis were RCTs, further confirming that statins are effective drugs for reducing the volume of atherosclerotic plaque in coronary arteries; (2) Meta-regressions showed that the percent change of CRP/hsCRP reduction was associated with a significant reduction in change of TAV/PV after statin therapy. After adjusting for percent change of LDL-C, age, gender and study duration, this association still existed. These findings indicate that the reduction in CRP/hsCRP levels might play an important role in the beneficial effects of statins on the progression of the atherosclerotic plaque. To the best of our knowledge, this study firstly investigated the association between CRP/hsCRP change and atherosclerotic plaque reduction using meta-regressions analyses.

Statins are HMG-COA reductase inhibitors. They reduce CHD incidence due to their lipid-regulating and extra-lipid-regulating effects and are important drugs for the primary and secondary prevention of CHD (32, 33). The benefits of statins have been demonstrated to be based on stabilization and/or reversal of atherosclerotic plaque (34–37). Particularly since the introduction of IVUS technology, numerous studies have used it as an important tool for studying coronary plaque. IVUS has recently become the main tool to study the effects of statins on coronary atherosclerotic plaque, and the data obtained by IVUS served as the primary endpoint in several studies (38, 39).

Recent studies suggest that LDL-C accumulates abnormally in the vascular wall due to endothelial cell dysfunction. In addition, LDL-C can be converted into oxidized low-density lipoprotein cholesterol (oxLDL-C), eventually promoting plaque progression (40). This implies that LDL-C change is a potential factor affecting plaque regression. A *post hoc* analysis found that statin therapy was associated with regression of coronary atherosclerosis when LDL-C was substantially reduced and high density lipoprotein cholesterol was increased by more than 7.5% (41). As a result, we separately included percent change of LDL-C as an independent variable to establish a simple linear regression model, and the results showed that LDL-C change did not influence the result. Moreover, when the percent change of CRP/hsCRP, percent change of LDL-C, age, gender and study duration were simultaneously taken as independent variables to establish the regression model, only the percent change of CRP/hsCRP had a significant impact on TAV/PV. These results indicated that in the included RCTs studies using statins as intervention drugs, the ability of statins to reduce TAV/PV is probably affected by their effect of reducing CRP/hsCRP instead of reducing LDL-C. The greater the reduction in CRP/hsCRP from baseline after statin treatment, the greater the reduction in TAV/PV. After adjusting for covariates (percent change of LDL-C, age, gender, and study duration), this association still existed. A previous study that analyzed the effect of pitavastatin treatment on changes of plaque volume had similar findings to our study. It demonstrated that TAV and PAV decreased more significantly in patients with reduction in hs-CRP ≥ 1 mg/dl than in those with reduction in hs-CRP < 1 mg/dl (42).

Various factors influence the degree of plaque regression under statin therapy. For instance, the statin drug type (43), plaque composition (44), and patient’s age and gender (45). In addition, clinical trials using IVUS demonstrated a linear relationship between LDL-C levels and reductions in atheroma burden under statin treatment (46). Despite the well-established causal role of LDL-C in the pathogenesis of atherosclerosis, our findings do not seem to support a reduction in TAV/PV relying on LDL-C levels. Recent investigations have demonstrated that changes in LDL-C levels are unrelated to plaque progression/regression following ezetimibe treatment (47). This is consistent with our research conclusions. However, the percent change of CRP/hsCRP was not significantly associated with SMD in change of PAV after adjusting for the percent change of LDL-C, age, gender and study duration. This could be because only seven trials were included in the regression analysis. The instability of research outcomes is caused by insufficient research data and an excessive number of independent variables.

It has previously been shown that anti-inflammatory therapy alone is beneficial for plaque regression (48). Considering the pleiotropic nature of statins, CRP/hsCRP is an important indicator of the anti-inflammatory effect of statins. Our findings imply that statins promote plaque regression, which is associated with their anti-inflammatory ability. And the effect of plaque regression may not be affected by their ability to regulate LDL-C.

At present, the main mechanisms of plaque formation include vascular endothelial dysfunction, intimal hyperplasia, lipid accumulation, and inflammatory response. Arterial inflammation plays an important role in the initiation and progression of atherosclerosis. Consistent with growing evidence that atherosclerosis is an inflammatory condition and many inflammatory cells, especially macrophages and foam cells can produce a variety of cytokines that may stimulate the hepatic expression of the CRP gene and up-regulate CRP production in the liver (49, 50). Therefore, elevated CRP, elevated hsCRP and changes of some other inflammatory markers may be potentially related to the risk of atherosclerosis development (50, 51). It is thought that the roles of CRP in the development of atherosclerotic plaque are complicated (52). Recent evidence propose that CRP and type oxidized LDL-C after being converted into foam cells stimulate tissue factor before thrombus formation, endothelial cell expression of adhesion molecules, and vascular endothelial dysfunction, all of which contribute to unstable atherosclerotic plaque (53, 54). In addition, several studies have suggested that atherosclerotic plaques also express CRP, and induce macrophage activation (55). Simultaneously, the expression and release of inflammatory factors are regulated to accelerate atherosclerotic plaque formation (56). Other studies also found that smooth muscle cells of atherosclerotic lesions could produce CRP and the locally produced CRP could participate in atherogenesis and the development of cardiovascular complications directly (50, 57). These associations between CRP and atherosclerosis suggest that inhibition of CRP may represent a therapeutic modality for the treatment of cardiovascular disease (49).

In addition to their cholesterol-lowering effects, recent clinical trials have established that the advantages of statins are based on their pleiotropic properties, such as reducing inflammation, stabilizing plaque, improving vascular endothelial function, suppressing vascular smooth muscle proliferation, and so on (58). And the ability to reduce inflammatory markers such as CRP and hsCRP is also included (59). Statins block CRP production by a variety of mechanisms (60). On the one hand, statins suppress CRP production by reducing IL-6, which is involved in stimulating CRP production by liver cells. On the other hand, statins reduce the production of inflammatory mediators from atherosclerotic plaques due to the decrease in LDL-C and consequently oxLDL-C (59, 61). Moreover, a direct interaction between statin molecules and CRP was found *in silico* evidence (62). Clinical trials also tried to confirm that the effects of statins on lowering CRP/hsCRP levels were beneficial to the prognosis of coronary plaque volume. For instance, an intervention trial evaluating rosuvastatin revealed that rosuvastatin reduced hs-CRP levels by 37% and hs-CRP are indicators of successful treatment with statins (63).

Despite the large body of evidence associating CRP with atherosclerotic lesions in previous studies, there is a lack of a direct correlation between its concentration and the extension of atherosclerosis as determined by imaging techniques (8). Our study indicates that the anti-inflammatory effects of statins may have a positive effect on atherosclerotic plaque regression as measured by the IVUS technique. This result suggests that CRP/hsCRP may be a potential therapeutic target in the process of atherosclerosis during statin therapy. Therefore, future research should continue to further study the effect of statin therapy on anti-inflammatory, including reducing serum CRP/hsCRP levels directly.

This study also has some limitations. First of all, we only searched 3 commonly used databases. It is possible that some studies in other databases and gray literature are overlooked. However, given that PubMed, EMBASE, and the Cochrane library are three most common databases used for meta-analysis and systematic review, our results should be a representative sample (64–66). Second, although the studies included in this meta-analysis were all RCTs and the quality of evidence was relatively higher, not all studies were double-blind trials. It is possible that performance bias is introduced. The meta-regression analysis (SMD in change of PAV as the dependent variable) was performed with 7 trials, which might lead to insufficient statistical power. In addition, this research adopted aggregate study-level data rather than individual-patient-level data. Individual-patient-level data may reflect the actual allocation plan of the subjects and improve the accuracy and integrity of the data. If future research could establish regression model based on individual-patient-level data to analyze the relationship between CRP/hsCRP levels and plaque regression, our research results could be further verified.

## Conclusion

In conclusion, our mete-analysis indicated that statins could significantly reduce plaque load measured by TAV/PV and PAV. Further meta-regression revealed that the percent change of CRP/hsCRP was significantly associated with the reduction in plaque volume. However, the percent change of LDL-C was not significantly associated with TAV/PV change or PAV change. Our results support that CRP/hsCRP decrease is crucial in the reduction of TAV/PV during statin treatment. Statins could promote plaque regression through their anti-inflammatory ability and that their ability to reduce plaque volume might be unaffected by their ability to reduce LDL-C. This finding will provide new avenues for future research on plaque regression.

## Data availability statement

The original contributions presented in this study are included in the article/Supplementary material, further inquiries can be directed to the corresponding author/s.

## Author contributions

RH, YM, and WX conceived and designed the study. RH, DJ, YM, CL, and SW performed the statistical analysis. DG, RH, and WX drafted and revised the manuscript. WX and QM were responsible for the integrity of the work as a whole. All authors contributed to the article and approved the submitted version.
