# Supplementary material for: C-reactive protein and coronary atheroma regression following statin therapy: A meta-regression of randomized controlled trials
Source: Front Cardiovasc Med. 2022 Nov 11;9:989527. doi: 10.3389/fcvm.2022.989527 (PMC9691666; doi:10.3389/fcvm.2022.989527)
Supplement: Supplementary file 2 [file Data_Sheet_2.docx]

#PAV

library(readxl)

library(meta)

# meta

getwd()

dta1<-read_excel("PAV.xlsx")

View(dta1)

m2 <- metacont(dta1$N_T, dta1$PAV_diff_T, dta1$PAV_diff_sd_T, dta1$N_C, dta1$PAV_diff_C, dta1$PAV_diff_sd_C,studlab = dta1$study,data = dta1, sm = "SMD",method.tau = "REML")

summary(m2)

forest(m2)

m2_reg_1<-metareg(m2,CRP_per_diff,intercept = T)

summary(m2_reg_1)

m2_reg_2<-metareg(m2,LDL_per_diff,intercept = T)

summary(m2_reg_2)

m2_reg_3<-metareg(m2,CRP_per_diff+LDL_per_diff,intercept = T)

summary(m2_reg_3)

m2_reg_5<-metareg(m2,duration,intercept = T)

summary(m2_reg_5)

m2_reg_4<-metareg(m2,CRP_per_diff+LDL_per_diff+Age_mean+Male_mean+duration,intercept = T)

summary(m2_reg_4)

#图片优化

# PAV

m2 <- metacont(dta1$N_T, dta1$PAV_diff_T, dta1$PAV_diff_sd_T, dta1$N_C, dta1$PAV_diff_C, dta1$PAV_diff_sd_C,studlab = dta1$study,data = dta1, sm = "SMD",fixed=F,random=T,method.tau = "REML")

summary(m2)

#jpeg("picture.jpeg",height=700,width=700)

forest(m2)

forest(m2,label.e = "Experimental", label.c = "Control",colgap.studlab="0.1cm",just.forest="center",label.left="favour [experimental]",label.right="favour [control]",

digits=2,digits.se=2,digits.tau2=2,digits.pval=2,family="sans",fontsize =10,lwd=2,col.diamond.fixed = "maroon",col.diamond.lines.fixed ="maroon",col.diamond.random = "maroon",

col.diamond.lines.random = "maroon",col.square = "skyblue",col.square.lines = "skyblue",col.study = "lightslategray",plotwidth="6cm",colgap.forest.left="0.2cm",colgap.forest.right="0.2cm",colgap.right="0.2cm",colgap.left ="0.2cm",just.forest="left")

#dev.off()

#######TAV

library(readxl)

library(meta)

# meta

getwd()

dta1<-read_excel("TAV.xlsx")

View(dta1)

# TAV

m1 <- metacont(dta1$N_T, dta1$TAV_diff_T, dta1$TAV_diff_sd_T, dta1$N_C, dta1$TAV_diff_C, dta1$TAV_diff_sd_C,studlab = dta1$study,data = dta1, sm = "SMD",method.tau = "REML")

summary(m1)

forest(m1)

m1_reg_1<-metareg(m1,CRP_per_diff,intercept = T)

summary(m1_reg_1)

m1_reg_2<-metareg(m1,LDL_per_diff,intercept = T)

summary(m1_reg_2)

m1_reg_3<-metareg(m1,CRP_per_diff+LDL_per_diff,intercept = T)

summary(m1_reg_3)

m1_reg_5<-metareg(m1,duration,intercept = T)

summary(m1_reg_5)

m1_reg_4<-metareg(m1,CRP_per_diff+LDL_per_diff+Age_mean+Male_mean+duration,intercept = T)

summary(m1_reg_4)

# PAV

getwd()

dta2<-read_excel("PAV.xlsx")

View(dta2)

m2 <- metacont(dta1$N_T, dta1$PAV_diff_T, dta1$PAV_diff_sd_T, dta1$N_C, dta1$PAV_diff_C, dta1$PAV_diff_sd_C,studlab = dta1$study,data = dta2, sm = "SMD",method.tau = "REML")

summary(m2)

forest(m2)

m2_reg_1<-metareg(m2,CRP_per_diff,intercept = T)

summary(m2_reg_1)

m2_reg_2<-metareg(m2,LDL_per_diff,intercept = T)

summary(m2_reg_2)

m2_reg_3<-metareg(m2,CRP_per_diff+LDL_per_diff,intercept = T)

summary(m2_reg_3)

m2_reg_5<-metareg(m2,duration,intercept = T)

summary(m2_reg_5)

m2_reg_4<-metareg(m2,CRP_per_diff+LDL_per_diff+Age_mean+Male_mean+duration,intercept = T)

summary(m2_reg_4)

metainf(m1,pooled = "random")

forest(metainf(m1,pooled = "random"))

funnel(m1)

funnel(m2)

metabias(m1, method.bias = "rank")

metabias(m1, method.bias = "linreg")

metabias(m2, method.bias = "rank")

metabias(m2, method.bias = "linreg")

# TAV

m1 <- metacont(dta1$N_T, dta1$TAV_diff_T, dta1$TAV_diff_sd_T, dta1$N_C, dta1$TAV_diff_C, dta1$TAV_diff_sd_C,studlab = dta1$study,data = dta1, sm = "SMD",fixed=F,random=T,method.tau = "REML")

summary(m1)

#jpeg("picture.jpeg",height=700,width=700)

forest(m1)

forest(m1,label.e = "Experimental", label.c = "Control",colgap.studlab="0.1cm",just.forest="center",label.left="favour [experimental]",label.right="favour [control]",

digits=2,digits.se=2,digits.tau2=2,digits.pval=2,family="sans",fontsize =10,lwd=2,col.diamond.fixed = "maroon",col.diamond.lines.fixed ="maroon",col.diamond.random = "maroon",

col.diamond.lines.random = "maroon",col.square = "skyblue",col.square.lines = "skyblue",col.study = "lightslategray",plotwidth="6cm",colgap.forest.left="0.2cm",colgap.forest.right="0.2cm",colgap.right="0.2cm",colgap.left ="0.2cm",just.forest="left")

dev.off()
